# Controllable Nanotribological Properties of Graphene Nanosheets

**DOI:** 10.1038/srep41891

**Published:** 2017-01-31

**Authors:** Xingzhong Zeng, Yitian Peng, Haojie Lang, Lei Liu

**Affiliations:** 1 College of Mechanical engineering, Donghua University, Shanghai 201620, China; 2 College of Mechanical engineering, Southeast University, Nanjing 211189, China

## Abstract

Graphene as one type of well-known solid lubricants possesses different nanotribological properties, due to the varied surface and structural characteristics caused by different preparation methods or post-processes. Graphene nanosheets with controllable surface wettability and structural defects were achieved by plasma treatment and thermal reduction. The nanotribological properties of graphene nanosheets were investigated using the calibrated atomic force microscopy. The friction force increases faster and faster with plasma treatment time, which results from the increase of surface wettability and the introduction of structural defects. Short-time plasma treatment increasing friction force is due to the enhancement of surface hydrophilicity. Longer-time plasma treatment increasing friction force can attribute to the combined effects of the enhanced surface hydrophilicity and the generated structural defects. The structural defects as a single factor also increase the friction force when the surface properties are unified by thermal reduction. The surface wettability and the nanotribological properties of plasma-treated graphene nanosheets can recover to its initial level over time. An improved spring model was proposed to elaborate the effects of surface wettability and structural defects on nanotribological properties at the atomic-scale.

Graphene derived from graphite is a highly attractive two-dimensional lubricating material, as well as a wear-resistant coating^1^,^2^,^3^. Its atomically thin lamellar structure and weak bonding between atomic layers by van der Waals bonds facilitate shear between adjacent layers, which is the origin of the low-friction characteristics^4^. Several different methods are available for the preparation of graphene, such as mechanical exfoliation, chemical vapor deposition (CVD) and thermal decomposition. The nanotribological properties of graphene prepared by different methods vary with the number of layers, the surface morphology and chemistry, and some other factors. For mechanically exfoliated and CVD graphene, the lower friction was observed as the number of graphene layers was increased, or when the stronger adhesion between the graphene and a high surface energy substrate was present, which both effects reduced the ability of graphene to pucker^4^,^5^. While for graphene produced by thermal decomposition, the mechanism for the dependence of the friction on the number of layers was proposed to be the electron-phonon coupling between the graphene and the underlying substrate^6^. In addition, the defects introduced in the process of growth or post-processing and the chemical modifications of graphene can increase friction^7^,^8^. Surface properties and structural characteristics of graphene are considered as two main factors affecting its nanotribological properties^9^,^10^.

Junfei Ou *et al*. prepared a hydrophobic trilayer film of APTES-GO-OTS and investigated its tribological behaviors. The low adhesion and the greatly reduced friction force were found on the film, which was attributed to the OTS-introduced surface hydrophobicity^11^. Tobin Filleter *et al*. studied the effect of structure on the nanotribology of ultrathin graphene oxide films with varying C/O ratio through friction force microscopy, and found higher C/O ratio GO exhibited much improved tribological properties and wear resistance which approached that of the graphene samples^12^. Young Jun Shin *et al*. discovered the friction coefficient of structural disordered graphene treated by oxygen plasma was larger than pristine graphene based on the micro-scale scratch tests^7^. Xiao-Yu Sun *et al*. investigated the friction behaviors between a diamond tip and three-layer graphene with single defect or stacked defects by using MD simulations, and showed that the friction force on defective graphite was significantly influenced by the defect location and type^10^. Many groups observed that the friction force increased to different degrees after graphene chemically modified with oxygen, fluorine or hydrogen and proposed various mechanisms to explain the increase of the friction force^8^,^13^,^14^,^15^,^16^.

The roles of surface properties and structural characteristics in nanotribological properties of graphene need to be further enriched, and the mechanism is also acquired to be understood more deeply. Moreover, their roles in nanotribological properties are always confused in previous studies. It is meaningful to control the nanotribological properties of graphene with controllable surface properties and structural defects. Plasma treatment is an effective way for the modification of surface properties and etching of graphene^17^,^18^,^19^,^20^. The structural defects, such as SP^3^ hybridization defects, vacancy-like defects and substitution-like defects, can be introduced into graphene by oxygen plasma treatment^21^,^22^. The graphene with controllable surface properties and structural defects can be obtained by appropriate plasma treatment.

The graphene nanosheets with controllable surface wettability and structural defects were achieved by plasma treatment and thermal reduction. The roles of surface wettability and structural defects in nanotribological properties were investigated separately using calibrated atomic force microscopy (AFM). The theoretical calculation and atomic model were adopted to uncover the underlying mechanism. The time-dependent nanotribological properties of plasma-treated graphene nanosheets were also explored.

## Results and Discussion

### Characterization of Graphene Nanosheets

**Figure 1:**
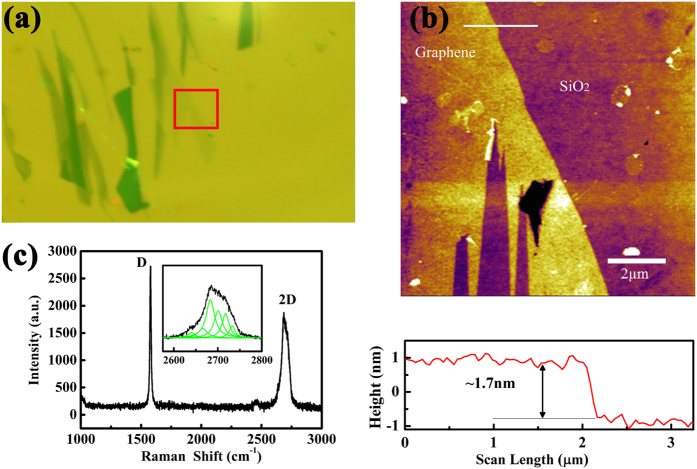
(**a**) Optical microscopy image, (**b**) AFM topographic image with cross-sectional height profile below, and (**c**) Raman spectra of the pristine graphene nanosheets. The red solid box in (**a**) is the location of topography imaging. The white solid line in (**b**) indicates the location where the cross-sectional profile is taken. The inset in (**c**) is the Lorenz multi-peaks fitting results from 2D peak in Raman spectra.

Figure 1(a) shows optical microscopy image of pristine graphene nanosheets. Topographic image obtained under the tapping mode of AFM is shown in Fig. 1(b). The thickness of the graphene nanosheets is about 1.7 nm from the cross-sectional height profile. Raman spectra are presented in Fig. 1(c). According to the Lorenz multi-peaks fitting, the 2D peak (2680 cm^−1^) consists of six components. On the basis of the height acquired from AFM topographic images and the number of sub-2D peaks fitted from Raman spectra, it can be concluded that the number of layers of graphene nanosheets is about three-layer^23^,^24^. In order to eliminate the influence of the thickness of graphene nanosheets on nanotribological properties, the thickness of all samples used in the experiments is ensured in the range of 1.5 to 2 nm.

A series of characterizations of graphene nanosheets with same thickness after plasma treatment are conducted by AFM, Raman spectroscopy and WCA measurements. The measured WCA values on the surface of graphene nanosheets before and after plasma treatment are shown in Table 1. The pristine graphene nanosheets are nearly hydrophobic (WCA = 89.5°), but plasma treatment lowers the WCA and makes them more hydrophilic. Meanwhile, the WCA decreases continuously as the treatment time increases, which demonstrates that plasma treatment can be used for obtaining the controllable surface wettability of graphene nanosheets.

**Figure 2:**
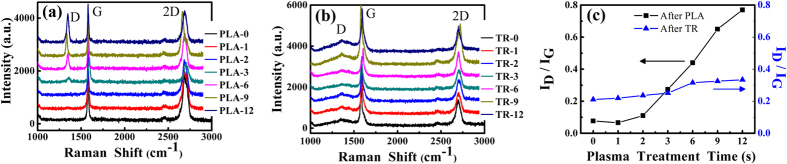
(**a**) Raman spectra of graphene nanosheets after plasma treatment, (**b**) Raman spectra of the plasma-treated graphene nanosheets after thermal reduction, (**c**) The intensity ratios between D peak and G peak of graphene nanosheets after plasma treatment and plasma-treated graphene nanosheets after thermal reduction as a function of plasma treatment time. Where PLA and TR represent plasma treatment and thermal reduction respectively, and the numbers after PLA and TR represent the plasma treatment time (s). The values of I_D_/I_G_ are concluded in Table S1.

Raman spectra of the plasma-treated and thermal-reduced graphene nanosheets are presented in Fig. 2(a) and (b) respectively. The intensity ratios between D peak and G peak as a function of plasma treatment time for different conditions are shown in Fig. 2(c). There are almost no D peaks (1350 cm^−1^) appeared after plasma treatment for 0 s, 1 s and 2 s, and the intensity ratio I_D_/I_G_ is also nearly zero. It can be concluded that plasma treatment for 1 s and 2 s do not destroy the structures of graphene nanosheets. When the treatment time reaches 3 s, the structural defects start to produce as D peak emerges, and since then the intensity of D peak and the intensity ratio I_D_/I_G_ increase continuously. In addition, the approximate linear relationship between I_D_/I_G_ and plasma treatment time is unexpected, which means that the degree of structural defects is proportional to the plasma treatment time.

Thermal reduction is widely used for the change of the surface properties via removing the hydrophilic functional groups, but also affects the structures inevitably. Structural disorders can be reduced by restoring SP^2^ networks of graphene, but the existing defects can also be extended and some new defects can be produced^25^. As shown in Fig. 2(b), the D peak appears on these thermal-reduced graphene nanosheets which do not be damaged by plasma treatment. Similar phenomenon can also be found from Fig. 2(c), where the intensity ratio I_D_/I_G_ of thermal-reduced graphene nanosheets is larger than that before thermal reduction. While for long-time plasma-treated graphene nanosheets, the intensity of D peaks and the intensity ratio I_D_/I_G_ get smaller after thermal reduction. These results suggest that the controllable surface properties can be obtained on short-time plasma-treated graphene nanosheets, while the controllable structural defects can be obtained on thermal-reduced graphene nanosheets.

In addition, the shapes of D peaks are different in both cases (Fig. 2(a,b)). The D peaks caused by plasma treatment are sharp and high, while become blunt and short after thermal reduction. The transformation forecasts the types and amounts of structural defects are different. Plasma treatment mainly creates SP^3^ hybridization defects because of the existence of oxygen-containing functional groups etc.^17^,^18^,^19^,^21^. Thermal reduction can develop new SP^2^ hybridization zones after carbon deoxidization and induce vacancy-like defects, since C atoms are also taken away during the removal of oxygen-containing functional groups^21^,^26^. Meanwhile, some defects induced by plasma are extended during thermal reduction^27^. It can be concluded that the degree of structural order lowers and the crystallite sizes diminish when the intensity ratio I_D_/I_G_ increases after thermal reduction.

### The Nanotribological Properties of Graphene Nanosheets after Plasma Treatment

**Figure 3:**
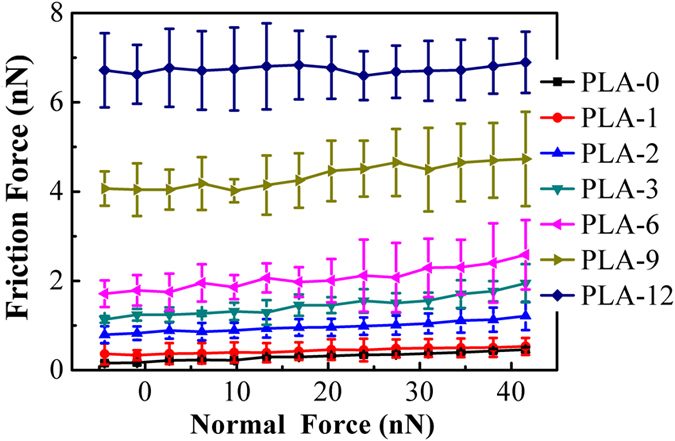
Friction force as a function of normal force measured on the graphene nanosheets after different time of plasma treatment.

Figure 3 shows the average friction force of graphene nanosheets as a function of normal force. The pristine graphene nanosheets exhibit the lowest friction force, and the friction force increases with the increase of plasma treatment time. Additionally, the growth rate of friction force also increases as the plasma treatment continues, especially after longer time of plasma treatment. When the plasma treatment time is relatively short, the enhancement of the surface hydrophilicity is the reason for the increase of the friction force. While for the long-time plasma treatment, the increase of the friction force and its growth rate is due to the combined effects between the further enhancement of the surface hydrophilicity and the continuous introduction of the structural defects.

**Figure 4:**
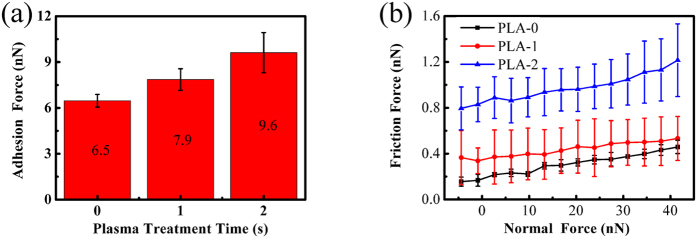
(**a**) The dependence of adhesion force on plasma treatment time, (**b**) The dependence of friction force on normal force on the graphene nanosheets after plasma treatment for 0 s, 1 s and 2 s. The numbers in (**a**) are the specific values of adhesion force.

Since no structural defects are induced by plasma treatment for 1 s and 2 s, only the surface properties of graphene nanosheets are changed. The effects of the controllable surface properties as a single factor on nanotribological properties can be performed on these plasma-treated graphene nanosheets. The dependences of adhesion force on plasma treatment time and friction force on normal force are summarized in Fig. 4(a) and (b) respectively. The adhesion force and friction force increase after plasma treatment, and continue to increase with more treatment time. In can be concluded that the nanotribological behaviors under this plasma treatment conditions are controlled by the enhancement of the surface hydrophilicity, as the decreased WCA shown (Table 1). But how the surface hydrophilicity is enhanced by plasma treatment?

The significance of water contact angle (WCA) is also reflected that it contains information about the surface energy of solid surfaces through the Young equation^28^:


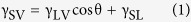


where γ_SV_, γ_LV_, γ_SL_ represent the solid surface free energy, liquid surface free energy, and solid-liquid interfacial energy respectively. θ is the contact angle between the solid surface and liquid. Referring to the value of γ_LV_ = 72.7 mJ/m^2^, γ_SL_ = 29 mJ/m^2^ 29,30 and the water contact angles measured above (Table 1), the surface energy of graphene nanosheets can be calculated using equation 1.

**Figure 5:**
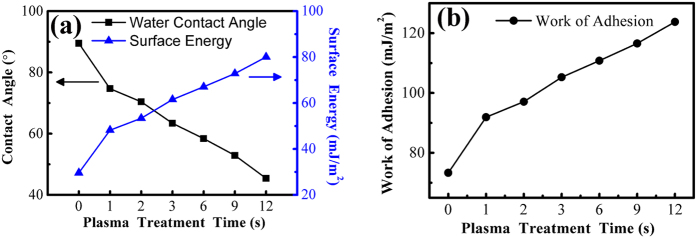
Water contact angle, surface energy (**a**) and work of adhesion (**b**) as a function of plasma treatment time on graphene nanosheets. The specific values of contact angle, surface energy and work of adhesion are concluded in Table S2.

Once the surface energy is acquired, the work of adhesion of graphene nanosheets can be obtained based on the equation^31^:


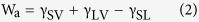


where W_a_ represents the work of adhesion. The work of adhesion can be used for approximatively characterizing the adhesion strength between the AFM tip and the graphene nanosheets. Larger work of adhesion corresponds to greater adhesion force. The calculated surface energy and corresponding water contact angle as a function of plasma treatment time are presented in Fig. 5(a), the obtained work of adhesion is shown in Fig. 5(b). The surface energy and work of adhesion increase continuously after plasma treatment, consistent with the adhesion force behaviors obtained in experiments (Fig. 4(a)). It indicates that the increase of the surface energy results in the enhanced surface hydrophilicity and the increased adhesion force.

**Figure 6:**
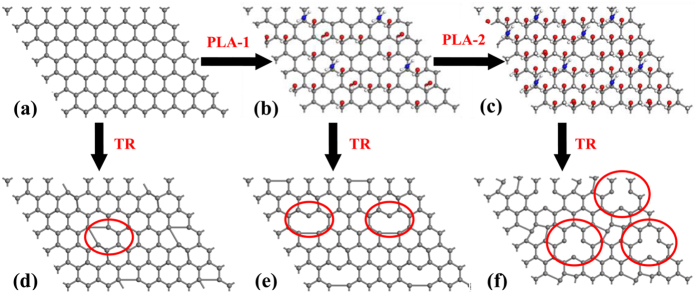
(**a**–**c**) Schematic illustrating the variation of surface functional groups after plasma treatment for 0 s, 1 s and 2 s respectively. (**d**–**f**) Schematic illustrating the variation of surface functional groups and structural defects after thermal reduction corresponds to (**a**–**c**). The gray ball, red ball, white ball and blue ball represent C atom, O atom, H atom and N atom respectively. TR and PLA represent thermal reduction and plasma treatment respectively. The number of balls denotes the degree of the change of surface properties. The areas circled by red ellipse are the location of part structural defects. Surface functional groups are removed and partial structural defects are created by thermal reduction.

The reasons for the increase of the surface energy after plasma treatment are concluded as follows. The nitrogen-containing and oxygen-containing functional groups are induced into the surface of graphene nanosheets by plasma^17^,^18^,^32^, and the amounts of functional groups add gradually with plasma treatment time. The schematic diagram of this process is established in Fig. 6, which colored balls represent these functional groups. The amounts of the balls increase continuously as the treatment time adds, which indicates the amounts of functional groups increase. The result is that the surface polarity enhances due to the removal of the non-polar functional group C-H and the accumulation of the polar functional groups C-O, O-C = O and C-N etc., which reinforces the hydrogen bonds and the dipole-dipole interactions in the vertical direction of the interface^33^,^34^. Moreover, owing to the experiments carried out in ambient conditions, chemical sorption as a result of the enhancement of the surface polarity brought in a water molecule monolayer, which raised water concentration of the surface^35^. All of these surface interactions increase the surface energy, and lead to the enhancement of the surface hydrophilicity of graphene nanosheets. In conclusion, plasma treatment introduces abundant functional groups, which leads to the increase of the surface energy. The increased surface energy enhances the surface hydrophilicity, which is reflected in the decreased WCA.

As for the increased adhesion force in Fig. 4(a). The adhesion force (F_po_) is generally a combination of the capillary force (F_cap_), the van der Waals force (F_vdW_), the electrostatic force (F_el_) and the forces due to chemical bonds (F_chem_)^35^. But in air conditions, the capillary force is considered to be the dominant contributor to adhesion force^36^. The enhanced surface hydrophilicity makes the sorption of water films on graphene surface much easier, which facilitates the growth of the water bridge between the AFM tip and the surface^37^, thereby increases the capillary force and adhesion force.

Theoretical calculation is used to quantitatively estimate the adhesion force between the AFM tip and the plasma-treated graphene nanosheets. The Johnson-Kendall-Roberts (JKR) theory for mechanical contacts between elastic solids is adopted to account for adhesion forces^38^. The relationship between the pull-off force (f_po_) and the work of adhesion (W_a_) per unit area measured at the pull-off point is


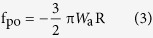


where R is the radius of curvature of the tip apex. While at the instability point where the tip is pulled from the surface, the contact radius is given by


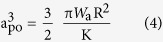


where K is the reduced modulus of the tip-sample contact. The reduced modulus is related to the Young’s modulus of the tip (E_t_) and sample (E_s_) materials:


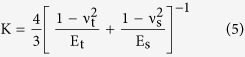


where ν_t_ and ν_s_ represent the respective Poisson ratios. The contact area can be written as 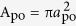. Consequently, the total pull-off force, namely the adhesion force, can be expressed as:


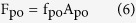


The assumed values of Young’s modulus and Poisson ratio for Si AFM tip are 130 Gpa and 0.27 respectively^39^. For graphene nanosheets, the corresponding values are 1000 Gpa and 0.15 respectively^40^. It should be noted that the calculated values for the adhesion force are approximate, as the tip radius (R) is associated with considerable uncertainties. Supposing the tip radius R~10 nm, equation 6 gave the adhesion force (F_po_) values of 3.83 nN, 5.59 nN and 6.13 nN for the graphene nanosheets after plasma treatment for 0 s, 1 s and 2 s respectively. These values are comparable to the adhesion forces acquired through experiments (Fig. 4(a)), which demonstrates that the experimental results are reliable.

As for the variation of the friction force after plasma treatment in Figs 3 and 4(b), it is likely that the increase of friction force is the result of the enhancement of adhesion. There is a correlation between adhesion force and friction force described by the Bowden-Tabor adhesion model for interfacial friction^41^. It states that the friction force is proportional to the true contact area (A) and to the shear strength (τ), as well as the second contribution which is referred to as ploughing force (F_p_). So the total friction force can be expressed as F_f_ = τA + F_p_. Since the shear strength scales with the adhesion force^42^ and the contact area have a positive correlation with the work of adhesion (see equation 4), the friction force increase simultaneously with the adhesion force.

Surface roughness is always considered as a factor increasing the friction force^14^. The increased surface energy and the enhanced surface hydrophilicity can adsorb some hydrocarbons and other impurities from air. The variation of the topographies after plasma treatment for 0 s, 1 s and 2 s is presented in Supplementary Figure S1. There is no significant difference occurred on the surface before and after plasma treatment. Roughness measurement was also performed on these topographies, but no significant contrasts were found. It indicates that the surface roughness is not dominant in our experiments. In addition, the influences of the AFM tip wear on the increase of the friction are also eliminated by using the same tip to measure the adhesion force and friction force before and after friction tests, as shown in Supplementary Figure S2.

**Figure 7:**
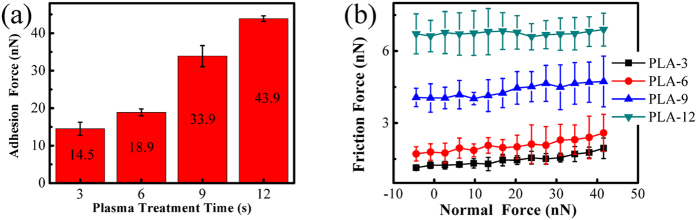
(**a**) The dependence of adhesion force on plasma treatment time. (**b**) The dependence of friction force on normal force on the graphene nanosheets after plasma treatment for 3 s, 6 s, 9 s and 12 s.

When long-time plasma treatments are performed on the graphene nanosheets, the variation of the adhesion force and friction force are displayed in Fig. 7. The adhesion force and friction force increase with the increase of the plasma treatment time. Meanwhile, the degree of the increase of the friction force becomes more and more significant with treatment time. The reasons that causing the adhesion force and friction force to increase depend on two factors. One of them is the variation of surface properties, the surface hydrophilicity is enhanced by plasma treatment. This can be determined from the WCA which diminishes with the increase of the plasma treatment time (Table 1). As discussed before, the enhancement of surface hydrophilicity is ascribed to the increase of the surface energy caused by the introduction of the oxygen-containing functional groups etc. The other is the generation of the structural defects. The Raman spectra in Fig. 2 illustrates that more defects are created after long-time plasma treatment.

**Figure 8:**
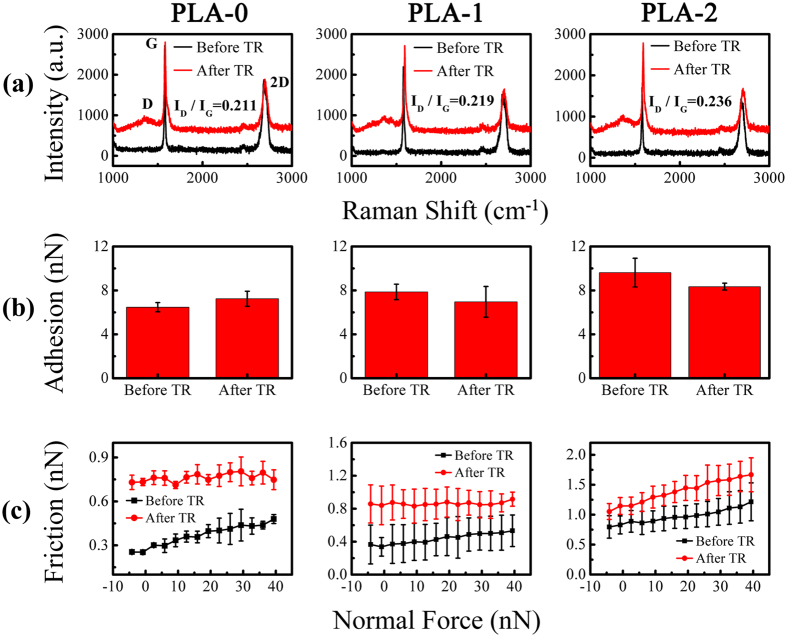
(**a**) Raman spectra of the plasma-treated graphene nanosheets before and after thermal reduction. (**b**) The comparison of adhesion force of the plasma-treated graphene nanosheets between before and after thermal reduction. (**c**) The comparison of friction force of the plasma-treated graphene nanosheets between before and after thermal reduction. Where PLA and TR represent plasma treatment and thermal reduction respectively, The numbers after PLA represent the plasma treatment time (s).

Thermal reduction can recover the surface properties and restore part of the structural defects, but it will also create new defects^25^,^43^,^44^,^45^. When thermal reduction is performed on the plasma-treated graphene nanosheets, the controllable structural defects can be a single factor to affect the nanotribological properties. Figure 8(a) shows the Raman spectra of the thermal-reduced graphene nanosheets treated by short-time plasma before. The D peak emerged after thermal reduction, G peak and 2D peak broadened slightly. The emergence of the D peak can be attributed to the structural disorder caused by the removal of intercalated water and oxygen-containing functional groups and the propagation of the nanosheets stacking disorder^25^,^27^. The decline of the crystallite size may be part of the reasons for Raman peaks broaden^44^,^46^. The surface properties of graphene nanosheets change from hydrophobic to hydrophilic after plasma treatment, but recover to hydrophobic after thermal reduction. Structural defects are created simultaneously by thermal reduction, and they are mainly vacancy-like defects as discussed in previous section. Meanwhile, the degree of structural defects caused by thermal reduction increases slightly with the plasma treatment time before, which indicated by the values of I_D_/I_G_ in Fig. 8(a).

As summarized in Fig. 8(b) and (c), the adhesion force of the graphene nanosheets without plasma treatment increases slightly after thermal reduction, while the adhesion force of the plasma-treated graphene nanosheets decreases marginally after thermal reduction. Nothing but these structural defects formed during thermal reduction are the reasons for the increase of the adhesion force. However, the decrease of the adhesion force is the result of the decrease of the surface hydrophilicity. The friction force increases invariably after thermal reduction regardless of the graphene nanosheets being treated by plasma or not before. This discrepancy between adhesion force and friction force illustrates that the effects of surface properties on adhesion force are greater than structural defects, but the effects on friction force are completely reverse. Meanwhile, the friction force of the thermal-reduced graphene nanosheets also increases with the increase of the plasma treatment time before (Fig. 8(c)). This can be understood by the increase of the degree of structural defects.

**Figure 9:**
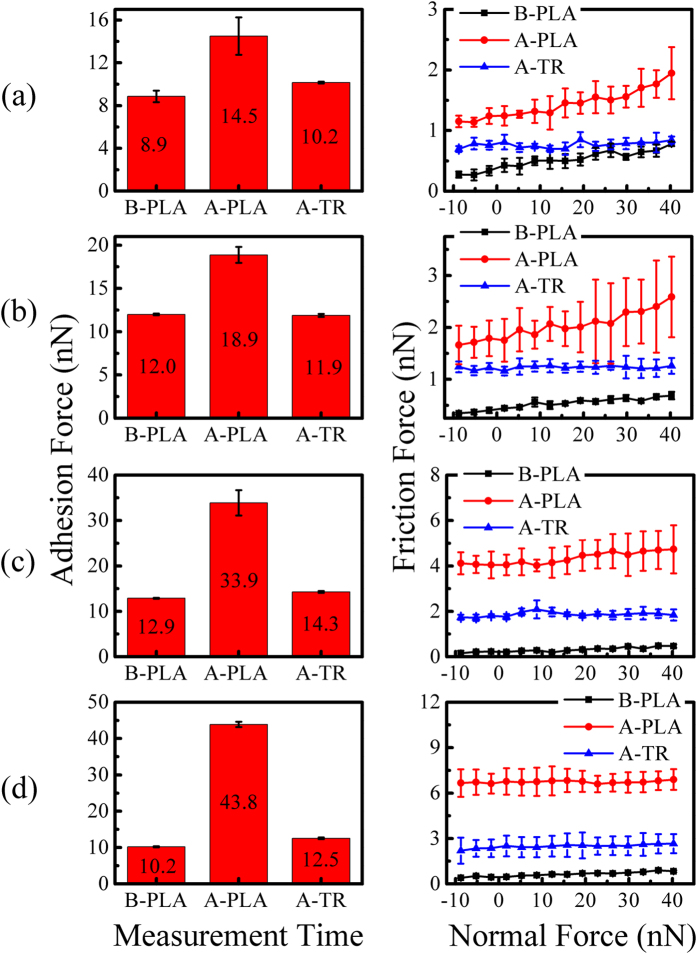
The adhesion force and friction force of the long plasma-treated graphene nanosheets before and after thermal reduction. The plasma treatment time are: (**a**) 3 s, (**b**) 6 s, (**c**) 9 s, and (**d**) 12 s respectively. Where PLA and TR represent plasma treatment and thermal reduction respectively, B and A are the abbreviation of before and after respectively.

When thermal reduction is performed on the long-time plasma-treated graphene nanosheets, structural defects decreases, but the total amounts of the residual and the new generated structural defects increase with the increase of plasma treatment time before thermal reduction (Fig. 2). The variation of the adhesion force and friction force after long-time plasma treatment and succeeding thermal reduction is concluded in Fig. 9. Thermal reduction reduces the increased adhesion force to a relatively low level. The friction force also decreases after thermal reduction, but its final values depend on the treatment time before thermal reduction. While the decrease of the adhesion force and friction force after thermal reduction can be ascribed to the recovery of the surface properties and the restoration of part structural defects.

**Figure 10:**
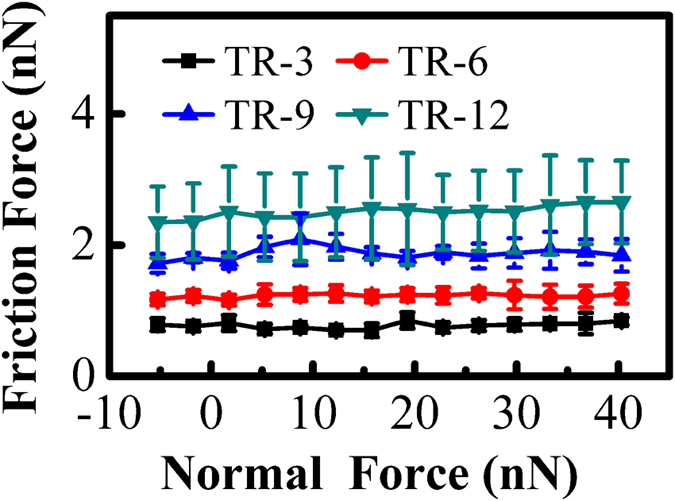
Friction force as a function of normal force on the long-time plasma treated graphene nanosheets after thermal reduction. Where TR represents thermal reduction, the numbers after TR represent the plasma treatment time (s) before thermal reduction.

The intensity ratio I_D_/I_G_ of thermal-reduced graphene nanosheets increases continuously with the increase of the plasma treatment time (Fig. 2(c)), which means the degree of defects increases correspondingly. Meanwhile, the surface properties of these graphene nanosheets recover to the same level via thermal reduction. So, the roles of the controllable structural defects in nanotribological properties can be explored again in these long-time plasma-treated graphene nanosheets after thermal reduction. Friction force as a function of normal force measured on theses thermal-reduced graphene nanosheets is summarized in Fig. 10. The friction force increases with the time of previous plasma treatment. The residual defects caused by plasma treatment and the regenerated defects via thermal reduction which include surface dislocation, vacancies, or corrugation are the origin of the increase of the friction force.

### The Recovery of Nanotribological Properties of Graphene Nanosheets

One of the major features of plasma treatment is the aging effect of the created surface modification^47^. The enhanced surface hydrophilicity by plasma treatment will weaken with time, which may recover the plasma-induced nanotribological properties of graphene nanosheets. In view of this, the WCA measurement was performed on the graphene nanosheets which treated by plasma for 10 s every two hours. The friction force was also obtained simultaneously through AFM.

**Figure 11:**
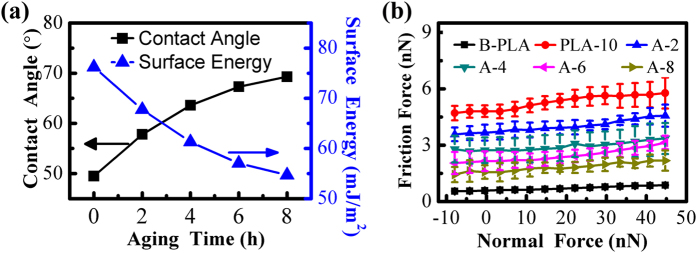
(**a**) The measured water contact angle (WCA) and the calculated surface energy as a function of aging time on the graphene nanoshets after plasma treatment for 10 s. (**b**) Friction force as a function of normal force on graphene nanosheets with different aging time. B and A are the abbreviation of before and after respectively. PLA represents plasma treatment. PLA-10 means the time of plasma treatment is 10 s. A-2, A-4, A-6 and A-8 represent the aging time after plasma treatment are 2 h, 4 h, 6 h and 8 h respectively. The values of the WCA and the surface energy are concluded in Table S3.

The measured WCA and the calculated surface energy (based on equation 1) every two hours are presented in Fig. 11(a). The WCA increases gradually with aging time, and levels off ultimately. While the surface energy displays an opposite tendency. It means the surface hydrophilicity weakens gradually, and the decrease of the surface energy is the reason for the weakening. The dependence of friction force on normal force under different aging time is shown in Fig. 11(b). The friction force increases after 10 s of plasma treatment, then decreases little by little with aging time, but the decrease rate slows down gradually. Meanwhile, the friction force has not returned to the initial level.

The recovery of the surface properties is due to the functional groups formed on the plasma-treated surface are not stable with time^48^. The surface loses its hydrophilic character gradually and back to the untreated state. But parts of structural defects created by plasma treatment can not be restored automatically. Thus the friction force decreases with aging time but can not recover to its initial value.

### The Atomic-scale Mechanism of Nanotribological Properties of Graphene Nanosheets

**Figure 12:**
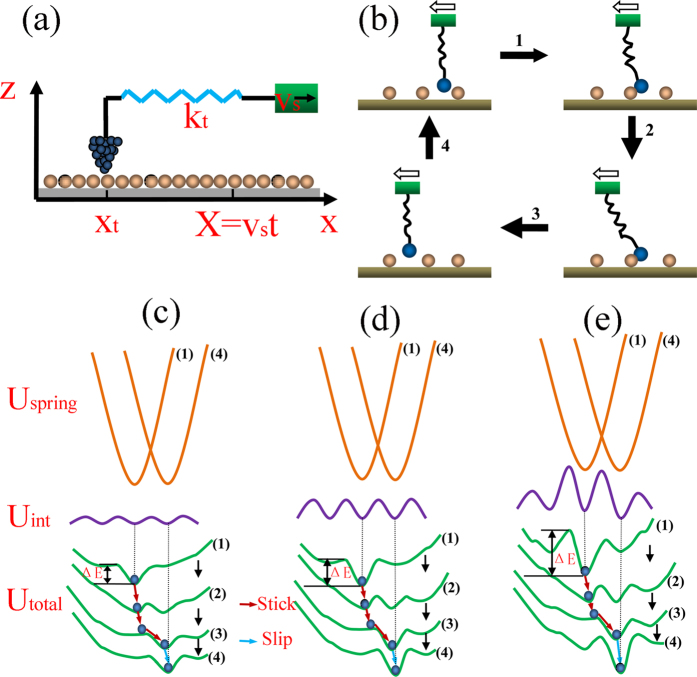
(**a**) FFM test system model. (**b**) The description of the movement of the tip in a atomic lattice period. The hollow arrows represent the sliding direction of the tip. (**c**–**e**) The variation of the potential and the tip atomic trajectory correspond to (**b**). Where (**c**,**d**) depict the weak and strong tip-surface interaction potential respectively, (**e**) depicts the tip-surface interaction potential affected by structural defects and (1–4) denote the time evolution of the potential by the tip scan. The fluctuation of U_int_ in (**e**) results from the structural defects. The brown solid lines represent the deformation energy of the spring (U_spring_), the purple solid lines represent the tip-surface interaction potential (U_int_), and the green solid lines represent the total potential energy of the system (U_total_). The blue dots are the locations of the tip atom in the total potential energy and ΔE is the potential barrier.

In order to investigate the inherent atomic-scale mechanism of the increase of friction force, a simple model representing the FFM test system is introduced in Fig. 12(a) and (b). A spring representing the cantilever in AFM uses to connect the tip with a rigid support, while the tip is considered to move relatively in a periodic potential field formed by its interaction with the graphene lattice. When support moves laterally at a rate of v_s_, the spring is gradually stretched and get deformation energy of 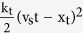 at the period of t, where x_t_ is the position of tip, k_t_ represents the equivalent spring constant. The total potential energy of the system can be expressed as^49^,^50^:


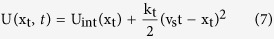


where U_int_(x_t_) refers to the interaction potential energy between the tip and the surface, which usually approximates by a sinusoidal form: 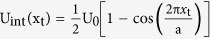. Here, U_0_ is the corrugation of the interaction potential (the height of the potential barriers) and a is the substrate (graphene) lattice constant.

According to the model mentioned above, the atomic-scale mechanism of the increase of friction force can be explained. Before the tip starts to move, its atoms are assumed to reside at the location of the minimum potential. Later, the deformation of the spring will increase continuously as the tip moves from right to left (Fig. 12(b)), because of the adhesion interaction between the tip and the surface. Until the deformation energy of spring overcomes the strongest adhesion, the tip atoms are triggered to generate high frequency vibration, and the tip is dragged to slip simultaneously. At this moment, the stored deformation energy of spring releases instantaneously in the form of heat, which causes frictional energy consumption^51^.

When the tip-surface interaction potential (U_int_) is weak, the variation of the potential barriers (ΔE) in total potential and the tip atomic trajectory correspond to Fig. 12(c). Conversely, the strong tip-surface interaction potential (U_int_) corresponds to Fig. 12(d). While the local variation of the tip-surface interaction potential (U_int_) caused by structural defects is shown in Fig. 12(e).

The adhesion force between the tip and the surface can change the tip-surface interaction potential (U_int_) and the deformation energy of spring (U_spring_). The greater the adhesion force, the stronger the tip-surface interaction potential, which more energy consumption is needed to overcome the larger potential barriers (Fig. 12(d)). However, if the adhesion force is weak, the total potential energy will follow the variation in Fig. 12(c), which only consumes less energy and leads to smaller friction force. In conclusion, the enhancement of the surface hydrophilicity caused by plasma treatment leads to the increase of the tip-surface interaction potential, which results in larger friction force.

The structural defects can strengthen the tip-surface interaction potential (U_int_) locally^52^,^53^. As shown in Fig. 12(e), the tip-surface interaction potential and the potential barriers at the locations of the defects will increase. Meanwhile, Larger defect concentration corresponds to wider range of the increase of the interaction potential. Thus more energy consumption is needed to conquer the potential barriers between the adjacent atomic lattices. Consequently, the friction force increases when structural defects produces, and the increase becomes faster and faster after the creation of more defects.

**Figure 13:**
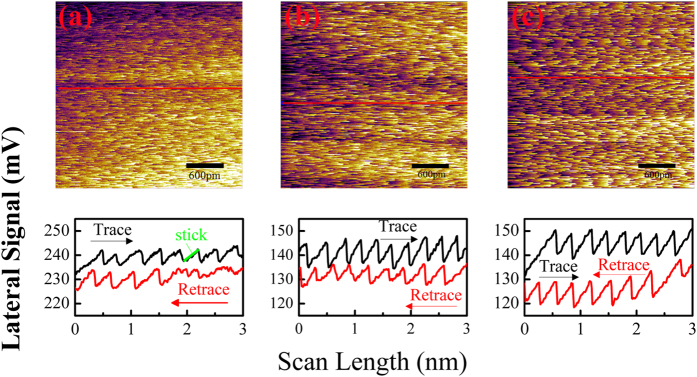
Lateral force maps and line profiles measured on graphene nanosheets. The plasma treatment time of graphene nanosheets are: (**a**) 0 s, (**b**) 1 s and (**c**) 2 s respectively. The red solid lines indicate the locations where the cross-sectional profiles are taken.

In addition, the adhesion-induced increase of friction force after plasma treatment can also be found from the lateral maps and lines profiles, as shown in Fig. 13. The saw-tooth fashion with the periodicity of the graphene surface lattice demonstrates the stick-slip movement of the tip on the surface of graphene nanosheets, which in accordance with the model introduced above. The enhanced adhesion caused by long plasma treatment leads to the increase of the difference between the trace line and retrace line as well as the length of the stick part as marked in Fig. 13, which means the friction force increases.

## Conclusions

The nanotribological properties of graphene nanosheets was controlled by surface wettability and structural defects obtained by plasma treatment and thermal reduction. For short-time plasma treatment, only the change of surface hydrophilicity increases the friction force. While for longer-time plasma treatment, both enhanced surface hydrophilicity and created structural defects increase the friction force. The structural defects also increase the friction force solely after the surface properties of graphene nanosheets are unified by thermal reduction. The friction force decreases gradually with aging time, but can not back to the initial value as a result of the residual structural defects.

The calculated adhesion force of the short-time plasma-treated graphene nanosheets based on Johnson-Kendall-Roberts (JKR) theory are comparable to the experimental values. An improved spring model was proposed to explain the increase of the friction force caused by the enhanced surface hydrophilicity and the generated structural defects. The enhanced adhesion between the tip and the surface increases the tip-surface interaction potential and the deformation energy of the spring, thus increases the friction force. The structural defects lead to the local improvement of the tip-surface interaction potential, further increase the friction force.

Our findings could provide insights in the reasonable design and use of graphene with controllable nanotribological properties as solid lubricants in nanomechanical applications. In addition, this work will propel the fundamental study of the nanotribology of grahene and help understand the underlying mechanism of the roles of surface properties and structural characteristics in nanotribological properties.

## Methods

The graphene nanosheets detached from the highly oriented pyrolytic graphite (HOPG) were deposited on a Si substrate with 300 nm thick oxide layer via the mechanical exfoliation method^54^. The exfoliated graphene nanosheets were treated with plasma for different time to change the surface wettability and create structural defects controllablly^17^,^18^,^19^,^46^. The power for plasma treatment was set to 6.5 W, and the treatment time was controlled to be 1 s, 2 s, 3 s, 6 s, 9 s and 12 s. Raman spectroscopy (inVia Reflex) using a 533 nm laser wavelength was applied to characterize the structural defects. The surface wettability was measured immediately by determining the water contact angle (WCA) with a deionized water droplet of volume 3 μl on the contact angle meter (OCA20). The values were the average of three repeated measurements, and the error was below 3°. Thermal reduction of graphene nanosheets was performed in the electric furnace (OTF-1200X) at 750 °C for 30 min under the mixture of nitrogen (100 sccm) and hydrogen (20 sccm).

The thickness of graphene nanosheets on the substrate was identified by optical microscopy (Motic/PSM 1000), AFM (MFP-3D, Asylum Research) and Raman spectroscopy. The silicon probes with a nominal normal spring constant of 3 N/m and tip radius less than 10 nm (Multi75Al-G, Budget Sensors) were used for imaging and friction tests. The adhesion force, friction force and stick slip phenomena of atomically thin graphene nanosheets were measured in ambient conditions (20–25 °C and 30–40% R.H). For quantitative force measurement, normal and lateral force calibrations of AFM tips were performed via noncontact method^55^. The adhesion force was determined by measuring the pull-off force which was the maximum force to pull the AFM tip out of contact with the surface. The error of the adhesion force was calculated as the standard deviation of five measurements recorded on different regions of the surface. Friction tests were performed by obtaining the friction loops in the areas of 300 nm × 300 nm with the scanning speed of 1 Hz. Each friction loop includes a complete trace and retrace scan over the same line. The friction force was related to the difference between the lateral forces obtained through trace and retrace scanning. The quantitative friction force was based on the average of the values measured through repeating the friction test three times. The applied normal force decreased stepwise until the tip detached from the surface, which zero value corresponded to the cantilever without bending in the normal direction.

## Additional Information

**How to cite this article**: Zeng, X. *et al*. Controllable Nanotribological Properties of Graphene Nanosheets. *Sci. Rep.* **7**, 41891; doi: 10.1038/srep41891 (2017).

**Publisher's note:** Springer Nature remains neutral with regard to jurisdictional claims in published maps and institutional affiliations.

## Supplementary Material

Supplementary Information

## Figures and Tables

**Table 1:** The water contact angle (WCA) on graphene nanosheets before and after plasma treatment.

Plasma treatment time (s)	0	1	2	3	6	9	12
WCA (°)	89.5	74.7	70.4	63.4	58.4	52.9	45.4
