# Supplementary material for: Controllable Nanotribological Properties of Graphene Nanosheets
Source: Sci Rep. 2017 Jan 31;7:41891. doi: 10.1038/srep41891 (PMC5282563; doi:10.1038/srep41891)
Supplement: Supplementary Information [file srep41891-s1.doc]

### Support Information for:

### Controllable Nanotribological Properties of Graphene Nanosheets

Xingzhong Zeng^1^, Yitian Peng^1,*^, Haojie Lang^1^, Lei Liu^2^

1. College of Mechanical engineering, Donghua University, Shanghai 201620, China

2. College of Mechanical engineering, Southeast University, Nanjing 211189, China

^*^Corresponding Author: [yitianpeng@dhu.edu.cn](mailto:yitianpeng@dhu.edu.cn)

# A. The values of the intensity ratio between D peak and G peak (I_D_ / I_G_)

Table S1 shows the values of the intensity ratio between D peak and G peak in Raman spectra. The intensity ratio I_D_ / I_G_ is nearly zero after plasma treatment for 0 s, 1 s and 2 s, which indicates that no defects are induced. The intensity ratio I_D_ / I_G_ begins to enlarge obviously after plasma treatment for 3 s, and it increases continuously with the increase of the plasma treatment time. While the intensity ratio I_D_ / I_G_ is incremental after thermal reduction, which means thermal reduction can create some structural defects.

## **Table S1. The intensity ratio of D peak and G peak.**

| Plasma treatment time (s) | I_D_ / I_G_ | |
| --- | --- | --- |
|  | After plasma treatment | After thermal reduction |
| 0 | 0.077 | 0.211 |
| 1 | 0.066 | 0.219 |
| 2 | 0.11 | 0.236 |
| 3 | 0.274 | 0.251 |
| 6 | 0.44 | 0.316 |
| 9 | 0.65 | 0.325 |
| 12 | 0.77 | 0.334 |

# B. The values of water contact angle, surface energy and work of adhesion

Table S2 shows the values of the measured water contact angle (WCA), the calculated surface energy and work of adhesion after different time of plasma treatment.

## **Table S2. The values of water contact angle, surface energy and work of adhesion.**

| Plasma treatment time  (s) | WCA(°) | Surface energy(mJ/m^2^) | Work of adhesion (mJ/m^2^) |
| --- | --- | --- | --- |
| 0 | 89.5 | 29.63 | 73.33 |
| 1 | 74.7 | 48.21 | 91.91 |
| 2 | 70.4 | 53.39 | 97.09 |
| 3 | 63.4 | 61.55 | 105.25 |
| 6 | 58.4 | 67.09 | 110.79 |
| 9 | 52.9 | 72.85 | 116.55 |
| 12 | 45.4 | 80.05 | 123.75 |

# C. The topographies and the measured surface roughness of graphene nanosheets

Figure S1 shows the AFM topographies after plasma treatment for 0 s, 1 s and 2 s respectively. No significant variations occur on the surface before and after plasma treatment. The surface roughness (Ra) was measured on the surface of the graphene nanosheets with 300 × 300 nm^2^ areas (red square in Figure S1). The measured surface roughness (Ra) of graphene nanosheets treated by plasma for 0 s, 1 s and 2 s was about 0.157 nm, 0.174 nm and 0.228 nm respectively.

Figure S1. AFM topographic images. (a) Without plasma treatment, (b) After plasma treatment for 1 s, (c) After plasma treatment for 2 s. The values in the figure represent the measured surface roughness (Ra). The red squares with 300 × 300 nm^2^ areas are the locations for the roughness measurement.


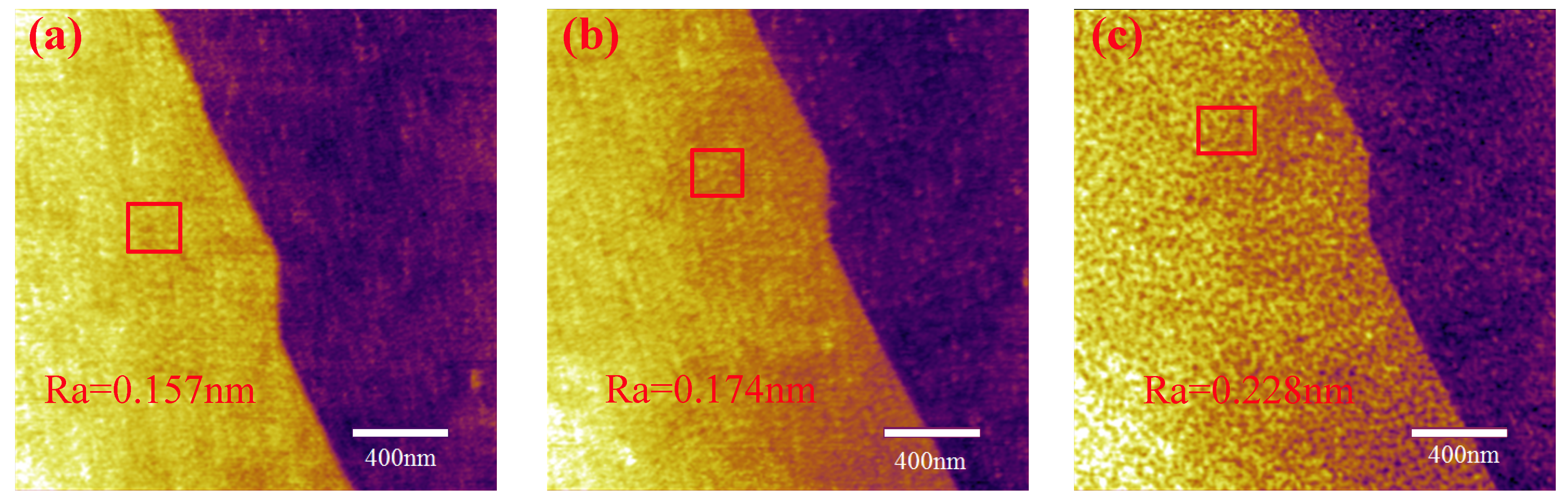


# D. The elimination of the effects of the wear of the AFM tip on nanotribological properties

In order to eliminate the effects of the wear of the AFM tip on nanotribological properties of graphene nanosheets, the adhesion force and friction force are measured on the same graphene surface before and after friction tests. Figure S2 shows the measured adhesion force and friction force, which are almost in the same level before and after friction tests. It indicates that the AFM tip is not wear after friction tests.

Figure S2. (a) Adhesion force measured on pristine graphene nanosheets before and after friction test, (b) Friction force as a function of normal force measured on pristine graphene nanosheets before and after friction tests.


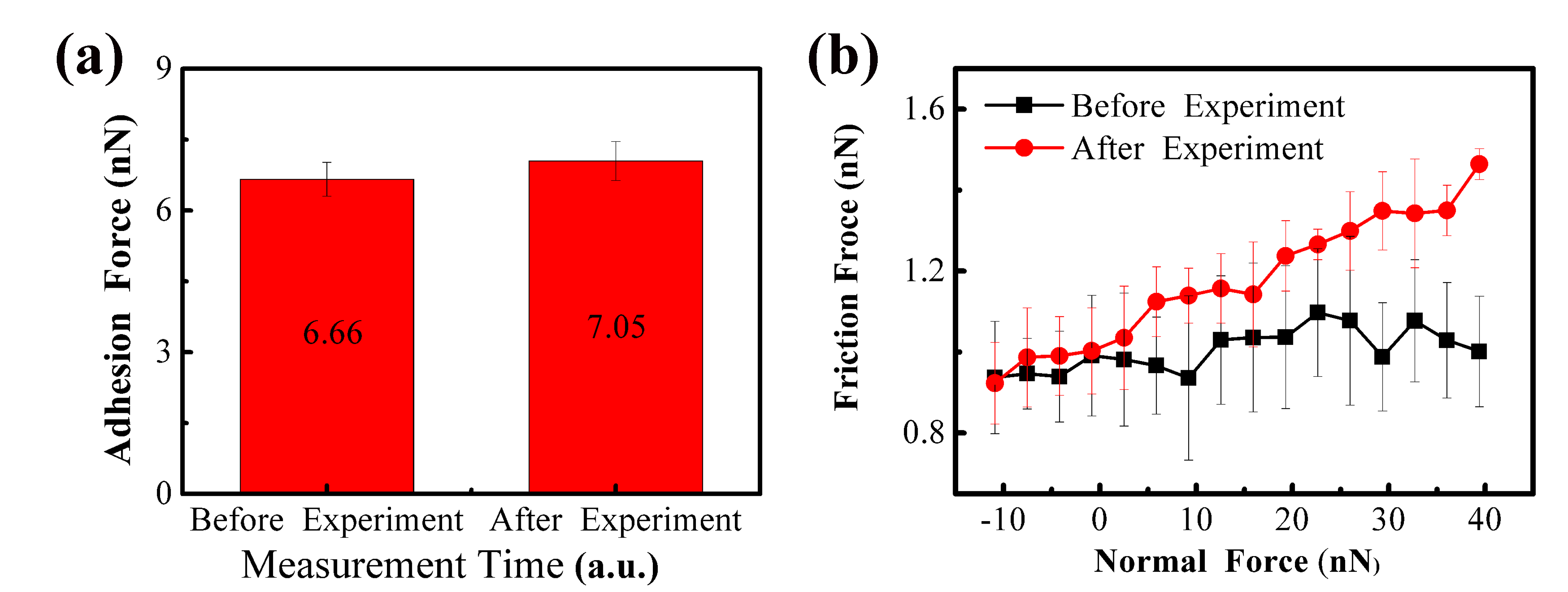


# E. The values of water contact angle and surface energy after different aging time

Table S3 shows the values of the measured water contact angle (WCA) and the calculated surface energy after different aging time, which the graphene nanosheets treated by plasma for 10 s before.

## **Table S3. The values of water contact angle and surface energy.**

| Aging time (h) | WCA (°) | surface energy (mJ/m^2^) |
| --- | --- | --- |
| 0 | 49.5 | 76.21 |
| 2 | 57.8 | 67.74 |
| 4 | 63.6 | 61.32 |
| 6 | 67.3 | 57.06 |
| 8 | 69.3 | 54.7 |
